# Cytoplasmic VDR expression as an independent risk factor for ovarian cancer

**DOI:** 10.1007/s00418-020-01894-6

**Published:** 2020-06-22

**Authors:** Bastian Czogalla, Eileen Deuster, Yue Liao, Doris Mayr, Elisa Schmoeckel, Cornelia Sattler, Thomas Kolben, Anna Hester, Sophie Fürst, Alexander Burges, Sven Mahner, Udo Jeschke, Fabian Trillsch

**Affiliations:** 1 Department of Obstetrics and Gynecology, University Hospital, LMU Munich, Marchioninistr 15, 81377 Munich, Germany; 2 Institute of Pathology, Faculty of Medicine, LMU Munich, Munich, Germany; 3 Department of Obstetrics and Gynecology, University Hospital Augsburg, Augsburg, Germany

**Keywords:** VDR, Vitamin D, Ovarian cancer, Risk factor, Immunohistochemistry

## Abstract

The vitamin D receptor (VDR), primarily known as a crucial mediator of calcium homeostasis and metabolism, has been shown to play a significant role in various cancer entities. Previous studies have focused on vitamin D and its receptor in gynecological cancers, noting that the receptor is upregulated in epithelial ovarian cancer (EOC). The aim of this study is to analyze the prognostic impact of VDR and its functional significance in ovarian cancer. Through immunohistochemistry, VDR staining was examined in 156 ovarian cancer samples. Evaluation of VDR staining was conducted in the nucleus and the cytoplasm using the semi-quantitative immunoreactive score, and the scores were classified into high- and low-level expressions. Expression levels were correlated with clinical and pathological parameters as well as with overall survival to assess for prognostic impact. Differences in cytoplasmic VDR expression were identified between the histological subtypes (*p* = 0.001). Serous, clear cell, and endometrioid subtypes showed the highest staining, while the mucinous subtype showed the lowest. Cytoplasmic VDR correlated with higher FIGO stage (*p* = 0.013; *Cc* = 0.203), positive lymph node status (*p* = 0.023; *Cc* = 0.236), high-grade serous histology (*p* = 0.000; *Cc* = 0.298) and grading from the distinct histological subtypes (*p* = 0.006; *Cc* = − 0.225). Nuclear VDR did not correlate with clinicopathological data. High cytoplasmic expression of VDR was associated with impaired overall survival (HR 2.218, 32.5 months vs. median not reached; *p* < 0.001) and was confirmed as a statistically independent prognostic factor in the Cox regression multivariate analysis. Additional knowledge of VDR as a biomarker and its interactions within the mitogen-activated protein kinase (MAPK) signaling pathway could potentially improve the prognosis of therapeutic approaches for specific subgroups in EOC.

## Introduction

Ovarian cancer is one of the most lethal tumor entities (Siegel et al. 2019). Insufficient screening methods and rising resistances to chemotherapy over the clinical course further contribute to the relatively low 5-year survival rate of around 45% (Baldwin et al. 2012; Siegel et al. 2019). Recommended therapy consists of cytoreductive surgery followed by adjuvant platinum-based chemotherapy combined with anti-angiogenic agents or followed by poly-ADP-ribose-polymerase inhibitors. To date, most reliable prognostic factors include the presence of residual disease after initial debulking surgery, the International Federation of Gynecology and Obstetrics (FIGO) stage, ascites volume, patient age, and histological subtype (Dembo et al. 1990; Vergote et al. 2001; Aletti et al. 2006; du Bois et al. 2009). However, widely accepted prognostic biomarkers are missing. Histologically, epithelial ovarian cancer (EOC) is classified into five main subtypes: high-grade serous, low-grade serous, mucinous, endometrioid, and clear cell histology, being distinguished in terms of phenotype, molecular background, and etiology (Kossaï et al. 2018). Considering the heterogeneity of ovarian cancer appears crucial for developing new prognostic and therapeutic strategies.

The VDR, as the receptor of the fat-soluble steroid vitamin D, known as 1ɑ,25(OH)_2_D_3_ or calcitriol, belongs to the superfamily of nuclear receptors. VDR regulates gene expression by binding to target genes with promoters containing a vitamin D response element (VDRE). VDR is a crucial mediator in calcium homeostasis and metabolism, inflammation, insulin-like growth factor signaling, and estrogen-related pathways and was identified in 30 different tissues (Valdivielso and Fernandez 2006; Holick and Chen 2008).

In recent years, increasing evidence suggests that vitamin D and VDR play a pivotal role in gynecological cancers (Deuster et al. 2017). VDR expression is increased in ovarian cancer and is associated with altered cancer cell proliferation in an interplay with other growth-stimulating factors like androgens (Ahonen et al. 2000; Villena-Heinsen et al. 2002; Friedrich et al. 2003; Anderson et al. 2006).

According to the increasing understanding of VDR`s role in ovarian cancer biology, expression analysis of VDR in different histological subtypes and of its correlation with survival was the primary aim in the current study.

## Materials and methods

### Patients and specimens

Ovarian cancer samples from 156 patients who underwent surgery for EOC at the Department of Obstetrics and Gynecology, Ludwig-Maximilian’s-University Munich from 1990 to 2002, were examined in this study. Clinical data were collected from the patients’ charts and follow-up data obtained from the Munich Cancer Registry. All specimens had been formalin fixed and paraffin embedded (FFPE). Patients with benign or borderline tumors were excluded and no patients had received neoadjuvant chemotherapy. Histological subtype and grading were determined by specialists at the Department of Pathology, Ludwig-Maximilian-University Munich. The staging was performed according to the WHO and FIGO Classification (2014). The clinicopathologic characteristics of the analyzed ovarian cancer patients are listed in Table 1.

**Table 1:** Clinicopathologic characteristics of the ovarian cancer patients

Clinicopathologic parameters	*n*	Percentage (%)
Histology		
Serous	110	70.5
Clear cell	12	7.7
Endometrioid	21	13.5
Mucinous	13	8.3
Primary tumor expansion		
TX	1	0.6
T1	40	25.6
T2	18	11.5
T3	97	62.3
Nodal status		
pNX	61	39.1
pN0	43	27.6
pN1	52	33.3
Distant metastasis		
pMX	147	94.2
pM0	3	1.9
pM1	6	3.8
Grading serous		
Low	24	21.8
High	80	72.7
Grading endometrioid		
G1	6	28.6
G2	5	23.8
G3	8	38.1
Grading mucinous		
G1	6	46.2
G2	6	46.2
G3	0	0
Grading clear cell		
G3	12	100.0
FIGO		
I	35	22.4
II	10	6.4
III	103	66.0
IV	3	1.9
Age		
≤ 60 years	83	53.2
> 60 years	73	46.8

### Ethical approval

The Ethics Committee of the Ludwig-Maximilians-University, Munich, Germany, approved this study (approval numbers 227-09 and 18-392). All tissue samples utilized for this investigation were collected from material from the archives of the Department of Obstetrics and Gynecology, University Hospital, LMU Munich, Munich, Germany, having initially been used for pathological diagnostics. The diagnostic procedures were concluded before the current study was conducted. During the analysis, the observers were fully blinded for patients’ data.

### Immunohistochemistry

Immunohistochemistry was completed as earlier outlined by our laboratory (Scholz et al. 2012). For the detection of VDR, FFPE tissue sections were dewaxed with xylol for 20 min, and later dehydrated in ascending concentrations of alcohol (70–100%). Afterward, they were exposed for epitope retrieval for 10 min in a pressure cooker using sodium citrate buffer (pH 6.0) holding 0.1 M citric acid and 0.1 M sodium citrate in distilled water. After cooling, the slides were cleaned in PBS twice. Endogenous peroxidase activity was quenched by dipping in 3% hydrogen peroxide (Merck, Darmstadt, Germany) and in methanol for 20 min. Non-specific binding of the primary antibodies was blocked by incubating the sections with “diluted normal serum” (10 ml PBS containing 150 μl horse serum; Vector Laboratories, CA) for 20 min at room temperature. Then, slides were incubated with the primary antibody [anti-vitamin D receptor antibody, mouse IgG, monoclonal, Serotec, Puchheim, Germany, clone 2F4 (MCA3543Z)] at room temperature for 60 min. After washing with PBS, slides were incubated in diluted biotinylated anti-serum secondary antibody (10 ml PBS containing 50 μl horse serum, Vector Laboratories, CA) for 30 min at room temperature. Following incubation with the avidin–biotin-peroxidase complex (diluted in 10 ml PBS, Vector Laboratories, CA) for 30 min and repeated PBS washing, visualization was conducted using substrate and chromagen 3,3′-diaminobenzidine (DAB; Dako, Glostrup, Denmark, catalog number K3468) for 8–10 min. Slides were then counterstained with Mayer’s acidic hematoxylin (Waldeck-Chroma, Münster, Germany, catalog number 2E-038) and dehydrated in an ascending series of alcohol followed by xylol. Negative and positive controls were employed to assess the specificity of the immunoreactions. Negative controls (colored in blue) were conducted in placental tissue by the replacement of the primary antibodies by species-specific (rabbit) isotype control antibodies (Dako, Glostrup, Denmark). For positive control, placental, vaginal and intestinal tissues were utilized.

### Staining evaluation

Specific VDR immunohistochemically staining reaction was observed in the nuclei and cytoplasm of the cells. The strength and distribution pattern of VDR staining was evaluated using the semi-quantitative immunoreactive score (IR score, Remmele’s score). To obtain the IR score result, the optional staining intensity (0 = no, 1 = weak, 2 = moderate, and 3 = strong staining) and the percentage of positive stained cells (0 = no staining, 1 ≤ 10% of the cells, 2 = 11–50% of the cells, 3 = 51–80% of the cells and 4 ≥ 81%) were multiplied. In all 156 (100%) EOC tissue samples, VDR staining was successfully performed. Cutoff scores for the IR scores were selected for the cytoplasmic VDR staining, taking into account the distribution pattern of IR scores in the collective. Cytoplasmic VDR staining was considered as low with IRS 0–2 and as high with IRS > 2.

For analyzing the images, the light microscope “Immunohistochemistry Type 307–148.001 512 686” by Leitz (Wetzlar, Germany) was used. The camera was produced by Fissler (IH-Camera 3CCD Colour Video Camera). For image acquisition, the software “Discuss Version 4,602,017-#233 (Carl C. Hilgers Technical Office) was used. Image bit depth: 24 mm; time and space resolution data: 760 + 574 pixel.

### Statistical analysis

SPSS 25.0 (v25, IBM, Armonk, New York) was used for statistical analysis. The distribution of clinical–pathological variables was assessed with the Chi-square test. The Mann–Whitney *U* test was utilized to compare IR scores of VDR between different clinical and pathological subgroups. Correlations between findings of immunohistochemical staining were measured using Spearman’s analysis. Through Kaplan–Meier (log-rank) estimates, survival times were analyzed. To identify an appropriate cutoff, the ROC curve was drawn, which is considered as one of the most reliable methods for cutoff point selection. In this context, the ROC curve is a plot representing sensitivity on the y-axis and (1-specificity) on the x-axis (Nakas et al. 2010). Consecutively, the Youden index, defined as the maximum (sensitivity + specificity-1) (Youden 1950), was used to find the optimal cutoff maximizing the sum of sensitivity and specificity (Fluss et al. 2005; Perkins and Schisterman 2006). For multivariate analyses, a Cox-regression model was used. *p* values less than 0.05 were considered to be significant.

## Results

### VDR expression correlates with clinical and pathological data

The clinicopathologic characteristics of the analyzed ovarian cancer patients are listed in Table 1. Out of 156 successfully stained ovarian cancer specimens, 153 (98%) showed positive nuclear VDR expression. In the cytoplasm, 154 (99%) cases were VDR positive. Median (range) immunoreactivity scores (IRS) for VDR in nuclei and cytoplasm were 3 (0.9) and 3 (0.8), respectively.

**Fig. 1:**
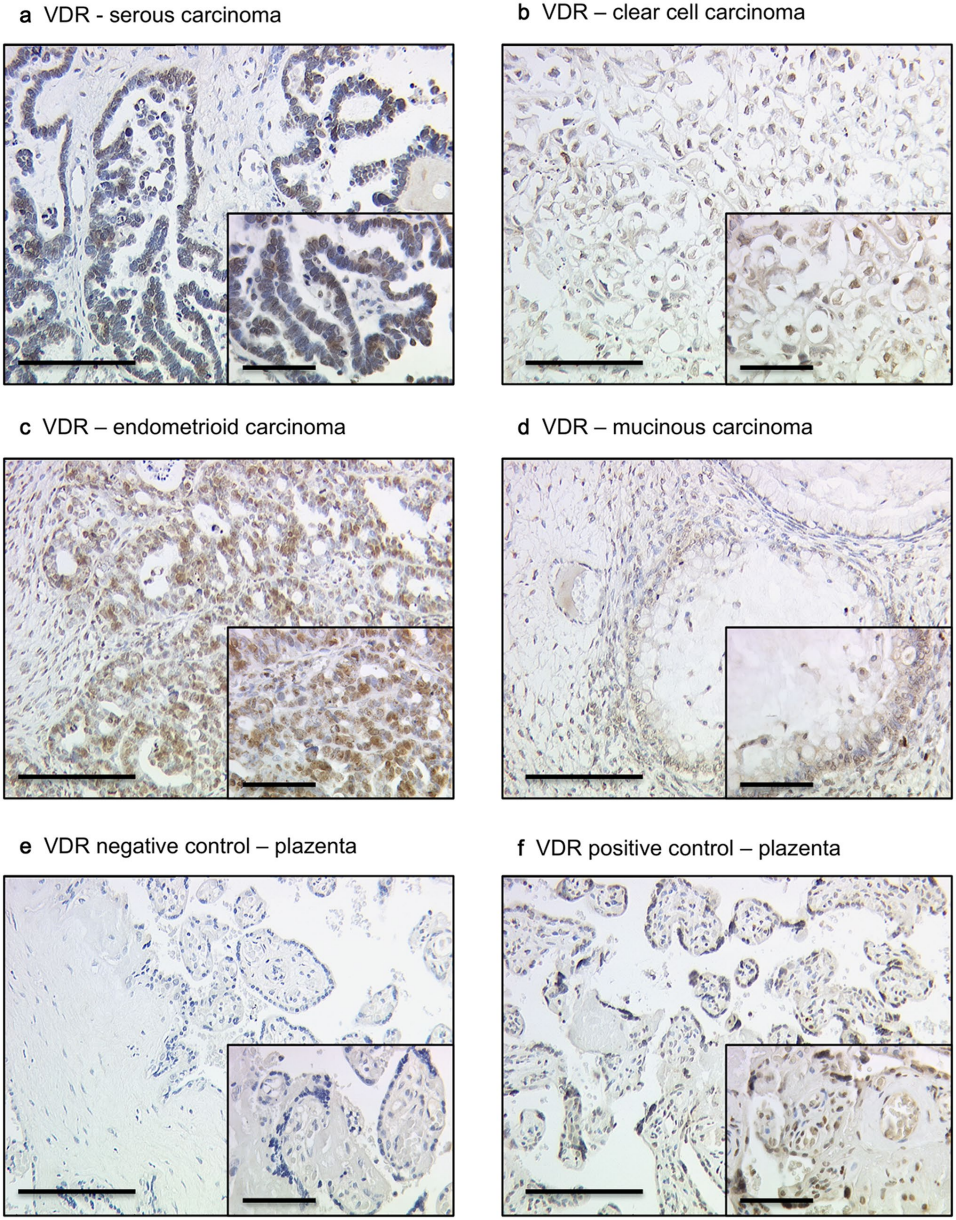
Detection of VDR with immunohistochemistry: **a** high VDR cytoplasm staining (IRS > 2) in ovarian cancer with serous, **b** clear cell, **c** endometrioid, (**d**) and mucinous histology. **e** VDR-negative control (**f**) and positive control in human placenta tissue. 10× (scale bar = 200 µm) and 25× (inserts, scale bar = 100 µm) magnification

Cytoplasmic VDR staining differed in the histological subtypes (*p* = 0.001): a high cytoplasmic VDR expression was found in serous, clear cell, and endometrioid histological subtypes, and a low VDR expression in the mucinous subtype (Fig. 1). In comparison, nuclear VDR expression did not show a significant difference between the histological subtypes (*p* > 0.05).

VDR expression displayed correlations to clinical and pathological data (Table 2). A positive correlation was observed between high cytoplasmic VDR staining and positive lymph node status (*p* = 0.023; *Cc* = 0.236) as well as with a higher FIGO stage (*p* = 0.013; *Cc* = 0.203). High cytoplasmic VDR staining correlated with high-grade serous histology (*p* = 0.000; *Cc* = 0.298), as well as with grading from the other histologic subtypes (*p* = 0.006; *Cc* = − 0.225). In the nucleus, VDR does not correlate with clinicopathological data. Nuclear VDR and cytoplasmic VDR expression were observed not to correlate with each other (*p* = 0.070; *Cc* = 0.147).

**Table 2:** Correlation between high cytoplasmic/ nuclear VDR expression and and clinicopathological data

	Cytoplasmic VDR expression		Nuclear VDR expression	
Variables	*p*	Correlation coefficient	*p*	Correlation coefficient
pT	0.133	0.122	0.653	-0.037
pN	0.023*	0.236	0.288	0.112
FIGO	0.013*	0.203	0.620	0.041
Grading				
Low-grade serous	0.121	− 0.125	0.065	0.150
High-grade serous	0.000**	0.298	0.155	− 0.116
Clear cell, endometrioid and mucinous-G1 to G3	0.006**	− 0.225	0.883	-0.012

Clinicopathologic data and VDR expression were correlated to each other using Spearman’s correlation analysis. Significant correlations are indicated by asterisks (*p < 0.05; **p < 0.01)

*p* two-tailed significance

### High cytoplasmic VDR expression is associated with impaired overall survival

**Fig. 2:**
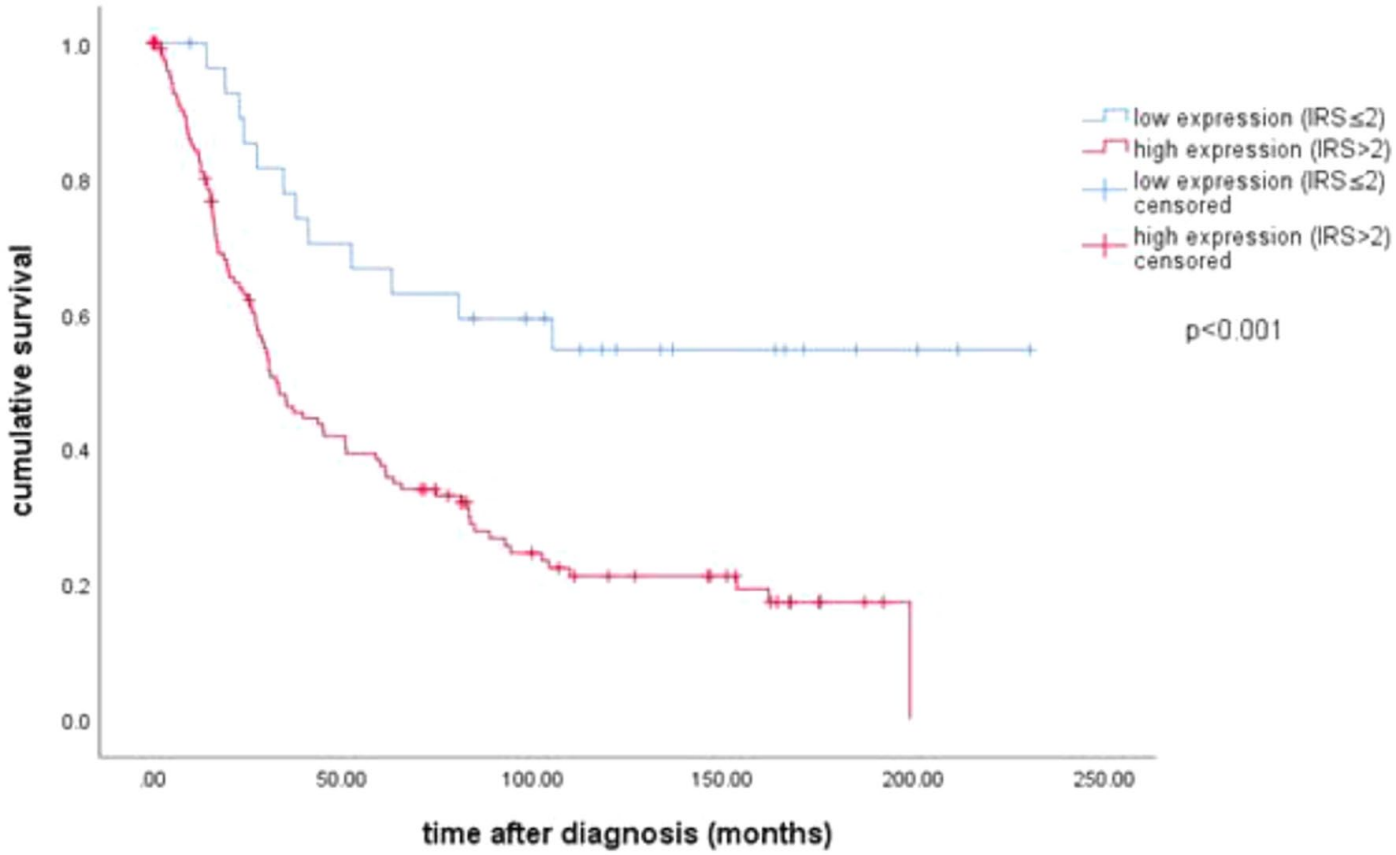
Kaplan–Meier estimate of cytoplasmic VDR: High cytoplasmic VDR expression (IRS > 2) was associated with impaired overall survival (HR 2.218, 32.5 months vs median not reached; *p* < 0.001)

The median age of the patients was 58.7 [standard deviation (SD) 31.4] years, with a range of 31–88 years. The median follow-up OS of the EOC patients was 34.4 (SD 57.8) months. Cytoplasmic VDR expression was significantly associated with a shorter OS (Fig. 2, 32.5 months vs. median not reached; *p* < 0.001).

### Cytoplasmic VDR and clinical/pathological parameters are independent prognostic factors

In a cox regression multivariate analysis of the present cohort with established prognostic markers, cytoplasmic VDR expression proved to be a statistically independent prognostic factor (HR 2.218, *p* = 0.025), as well as cancer grading (HR 1.604, *p* < 0.001), FIGO stage (HR 1.947, *p* < 0.001) and patient’s age (HR 1.628, *p* = 0.019) (Table 3) were proved independent factors. Conversely, the prognostic impact of histological subtypes was not confirmed to be of independent significance.

**Table 3:** Multivariate analysis

Covariate	Coefficient	Hazard ratio	95% CI		*p* value
			Lower	Upper	
Histology (serous vs other)	− 0.037	0.963	0.637	1.457	0.86
Grading (low vs high)	0.472	1.604	1.158	2.138	0.000**
FIGO (I, II vs III, IV)	0.666	1.947	1.409	2.690	0.000**
Patients’ age (≤ 60 vs > 60 years)	0.487	1.628	1.082	2.449	0.019*
VDR cytoplasmic	0.797	2.218	1.106	4.448	0.025*

A multivariate Cox regression model was established to investigate independency of prognostic factors. Significant independent factors are indicated by asterisks (*p < 0.05; **p < 0.01)

*CI* confidence interval

## Discussion

This present study focused on VDR expression in different histologic subtypes of EOC and its correlation with clinicopathological parameters. Patients with an increased cytoplasmic VDR expression were confirmed to have a significantly impaired OS. Moreover, cytoplasmic VDR expression was identified as an independent prognostic factor for OS; therefore, these results suggest a functional role of VDR in ovarian cancerogenesis, which merits further investigations.

VDR has been traditionally considered as the nuclear receptor of vitamin D with a crucial role in calcium homeostasis and metabolism (Holick and Chen 2008). However, increasing evidence suggests that vitamin D and VDR play a pivotal role in the development of ovarian cancer. Epidemiological studies showed a reduced ovarian cancer risk in southern countries, indicating an association with the inhibition of vitamin D synthesis (Garland et al. 2006). Moreover, studies demonstrated an association between low circulating 25-hydroxyvitamin D, a prehormone of vitamin D, and a higher ovarian cancer incidence (Bakhru et al. 2010; Yin et al. 2011; Walentowicz-Sadlecka et al. 2012; Anastasi et al. 2016; Ong et al. 2016), while increased 25-hydroxyvitamin D levels at diagnosis seem to be associated with longer overall survival in ovarian cancer patients (Webb et al. 2015). 25-Hydroxyvitamin D reduces proliferation and induces cell cycle arrest of ovarian cancer cells as well as in animal models (Jiang et al. 2003; Zhang et al. 2005; Thill et al. 2015). Furthermore, vitamin D is involved in epithelial–mesenchymal transition (EMT), a crucial process in cancerogenesis and tumor progression. Vitamin D decreases the expression of relevant transcription factors of EMT and thereby reduces migration and invasion of SKOV-3 cells. This might explain the effect of vitamin D on ovarian cancer in vitro and in vivo (Hou et al. 2016).

VDR is expressed in both benign and malignant ovarian tissues and influences the ovarian function by mediating estrogen biosynthesis and aromatase gene expression (Lurie et al. 2007). In vivo studies demonstrated that VDR-null mice show gonadal insufficiency and low aromatase activity.

Our findings are in line with previous reports showing that VDR expression is increased in ovarian cancer (Villena-Heinsen et al. 2002; Friedrich et al. 2003; Anderson et al. 2006; Agic et al. 2007). In 2010, Silvagno et al. analyzed the correlation between VDR expression and clinicopathological parameters, reporting predominantly cytoplasmic staining for VDR in ovarian cancer tissue (Silvagno et al. 2010). A comparable cytoplasmic staining pattern of VDR was noticed in malignant melanoma, colon and vulvar cancer affecting tumor progression and prognosis (Matusiak et al. 2005; Salehin et al. 2012; Hutchinson et al. 2018). However, the functional effect of cytoplasmic VDR cannot be described by its nuclear signal cascade. Interestingly, VDR may mediate its molecular effect through two different pathways. While the classic nuclear pathway involves genes with promoters containing a vitamin D response element (VDRE) and consecutively regulates gene expression, the non-nuclear VDR-mediated pathway follows different mechanisms: VDR interacts with c-Src in the plasma membrane activating c-RAF and subsequently the MEK1/2/ERK1/2 pathway (Cordes et al. 2006; Buitrago and Boland 2010; Han et al. 2010; Doroudi et al. 2014). The extracellular signal-regulated kinase (ERK) pathway is one of the major signaling cascades of the MAPK signaling pathway, which plays a crucial role in cancerogenesis, including cell proliferation, differentiation, migration, apoptosis, and chemoresistance. Preclinical and early clinical studies underline the relevance of the ERK/MAPK pathway in ovarian cancer (Hsu et al. 2004; Bartholomeusz et al. 2006; Al-Ayoubi et al. 2008; Ohta et al. 2009; Vergara et al. 2012; Chang et al. 2012; Fujisawa et al. 2012; Wang et al. 2014; Su et al. 2016; Bai et al. 2016; Ma et al. 2018). In this context, high MAPK activity in tumors was associated with high cytoplasmic VDR expression, indicating an association of these pathways, which could lead to a crucial role of the non-nuclear VDR pathway in cancerogenesis. Hutchinson et al. showed a similar interaction in malignant melanoma (Hutchinson et al. 2018). Decreased nuclear and high cytoplasmic VDR expressions were associated with malignant progression in terms of dermal invasion and metastasis. Malignant melanomas that retained exclusive nuclear VDR at the tumor base did not metastasize in this study. Furthermore, high MAPK activity in tumors expressing cytoplasmic VDR was associated with increased cell growth and worsened prognosis. In contrast, MAPK inhibition produced nuclear migration of VDR and decreased cell viability in vitro.

Inhibition of the MAPK pathway in EOC has been considered as a potential approach in subgroups of patients with specific histologic profiles, especially low-grade histology, although targeted therapies have so far failed to exhibit reliable therapeutic effects (Farley et al. 2013; Han et al. 2018; Fernandez et al. 2019). Thus defining subgroups of suitable patients for MAPK inhibition remains highly important.

Our data suggest cytoplasmic VDR as a potential predictive biomarker that might distinguish between tumors which are MAPK inhibitor sensitive and those which are not. Thus, cytoplasmatic VDR could be used to predict which patients profit from an MAPK inhibitor therapy.

Nevertheless, further studies are needed to prove our hypothesis as a basis for a better understanding of therapy in EOC subgroups.

## Data Availability

The datasets generated and/or analyzed during the current study are available from the corresponding author upon reasonable request. Data are available from the corresponding author on reasonable request.

## Abbreviations

Cc: Correlation coefficient
CI: Confidence interval
EMT: Epithelial–mesenchymal transition
EOC: Epithelial ovarian cancer
ERK: Extracellular-signal-regulated kinase
FFPE: Formalin fixed and paraffin embedded
FIGO: Fédération Internationale de Gynécologie et d'Obstétrique
IRS: Immunoreactive score
MAPK: Mitogen-activated protein kinase
OS: Overall survival
SD: Standard deviation
VDR: Vitamin D receptor VDR
VDRE: Vitamin D response element
WHO: Word Health Organization
